# The Role of Phosphorus Limitation in Shaping Soil Bacterial Communities and Their Metabolic Capabilities

**DOI:** 10.1128/mBio.01718-20

**Published:** 2020-10-27

**Authors:** Angela M. Oliverio, Andrew Bissett, Krista McGuire, Kristin Saltonstall, Benjamin L. Turner, Noah Fierer

**Affiliations:** a Department of Ecology and Evolutionary Biology, University of Colorado, Boulder, Colorado, USA; b Cooperative Institute for Research in Environmental Sciences, University of Colorado, Boulder, Colorado, USA; c CSIRO Oceans and Atmosphere, Hobart, TAS, Australia; d Institute of Ecology and Evolution, University of Oregon, Eugene, Oregon, USA; e Smithsonian Tropical Research Institute, Balboa, Ancon, Republic of Panama; University of Georgia

**Keywords:** phosphorus limitation, C-P lyase pathway, organophosphonate degradation, phosphate starvation, soil microbiology

## Abstract

Phosphorus (P) is an essential nutrient that is often in limited supply, with P availability constraining biomass production in many terrestrial ecosystems. Despite decades of work on plant responses to P deficiency and the importance of soil microbes to terrestrial ecosystem processes, how soil microbes respond to, and cope with, P deficiencies remains poorly understood. We studied 583 soils from two independent sample sets that each span broad natural gradients in extractable soil P and collectively represent diverse biomes, including tropical forests, temperate grasslands, and arid shrublands. We paired marker gene and shotgun metagenomic analyses to determine how soil bacterial and archaeal communities respond to differences in soil P availability and to detect corresponding shifts in functional attributes. We identified microbial taxa that are consistently responsive to extractable soil P, with those taxa found in low P soils being more likely to have traits typical of oligotrophic life history strategies. Using environmental niche modeling of genes and gene pathways, we found an enriched abundance of key genes in low P soils linked to the carbon-phosphorus (C-P) lyase and phosphonotase degradation pathways, along with key components of the high-affinity phosphate-specific transporter (Pst) and phosphate regulon (Pho) systems. Taken together, these analyses suggest that catabolism of phosphonates is an important strategy used by bacteria to scavenge phosphate in P-limited soils. Surprisingly, these same pathways are important for bacterial growth in P-limited marine waters, highlighting the shared metabolic strategies used by both terrestrial and marine microbes to cope with P limitation.

## Abstract (precis)

Phosphorus (P) is an essential nutrient that is often in limited supply, with P availability constraining biomass production in many terrestrial ecosystems. Despite decades of work on plant responses to P deficiency and the importance of soil microbes to terrestrial ecosystem processes, how soil microbes respond to, and cope with, P deficiencies remains poorly understood. We studied 583 soils from two independent sample sets that each span broad natural gradients in extractable soil P and collectively represent diverse biomes, including tropical forests, temperate grasslands, and arid shrublands.

## INTRODUCTION

Phosphorus (P) is an essential nutrient for the growth of all organisms; yet, it is a scarce resource in many ecosystems (1, 2). In terrestrial soils, P frequently limits the growth of plants and microbes, with the majority of soil P associated with recalcitrant or insoluble compounds that are not readily available for biological uptake (3, 4). Plant-available P generally decreases during ecosystem and soil development as P becomes less available with occluded P (i.e., P that is physically bound by minerals) and soil organic P typically becoming the dominant pools of P (5). Thus, soil P has a major role in constraining biomass accumulation and primary productivity in many systems, particularly in tropical forests and agricultural systems with highly weathered soils (6). Microbes play an important role in altering soil P availability, both by increasing plant-available P through mineralization and solubilization and, conversely, by competing for available P with other organisms, including plants (7). Thus, characterizing how belowground microbial communities shift in response to differences in P availability is critical for understanding soil P dynamics.

In marine systems, bacteria can cope with P limitation through a variety of mechanisms, including utilization of phosphonates (8, 9) and nonphosphorus lipid renovation (1, 10). In contrast, we know far less about soil microbial responses to P limitation, even though P availability is presumed to be low in many soils (3). Most of our knowledge of soil microbial responses to differences in P availability come from the characterization of bacterial isolates under highly controlled lab settings (11, 12). Such work has been used to identify specific strains, metabolic pathways, and regulatory systems that are enhanced under low P or high P conditions. For example, bacterial isolates from soil have been evaluated for their ability to grow on different P sources, with many of these isolates capable of growing on a wide range of compounds to obtain phosphate (11, 13), including inositol phosphate (via acid or alkaline phosphatase enzymes), phosphites, and organophosphonates (via enzymes that cleave C-P bonds). However, it remains unclear if these insights gained from *in vitro* studies of bacterial isolates are applicable to understanding the *in situ* microbial responses to differing levels of P availability. Specifically, we have a limited understanding of microbial P metabolism in natural soil ecosystems, the traits associated with survival and growth under P-limited conditions, or even how the taxonomic composition of soil microbial communities is altered by differences in P availability.

There are a number of reasons why these knowledge gaps persist. First, although measures of P availability have been around for a long time, it remains difficult to apply them in natural ecosystems where organic P turnover represents a major contribution to P nutrition (14). Second, much of the previous work on microbial responses to differences in soil P have relied on fertilization experiments (15, 16) where P availability is elevated by adding exogenous inputs of mineral P (typically high levels of calcium phosphate). These P dynamics likely do not reflect the conditions experienced by most organisms in natural ecosystems (17). Third, previous studies conducted on naturally occurring gradients in P availability have typically focused on only a few sites (18, 19). From this work, we know that lower P availability is associated with efficient phosphate uptake/transport systems and P starvation response regulation, but we do not know if these observed patterns are generalizable across a range of different soils and geographic locations.

**FIG 1:**
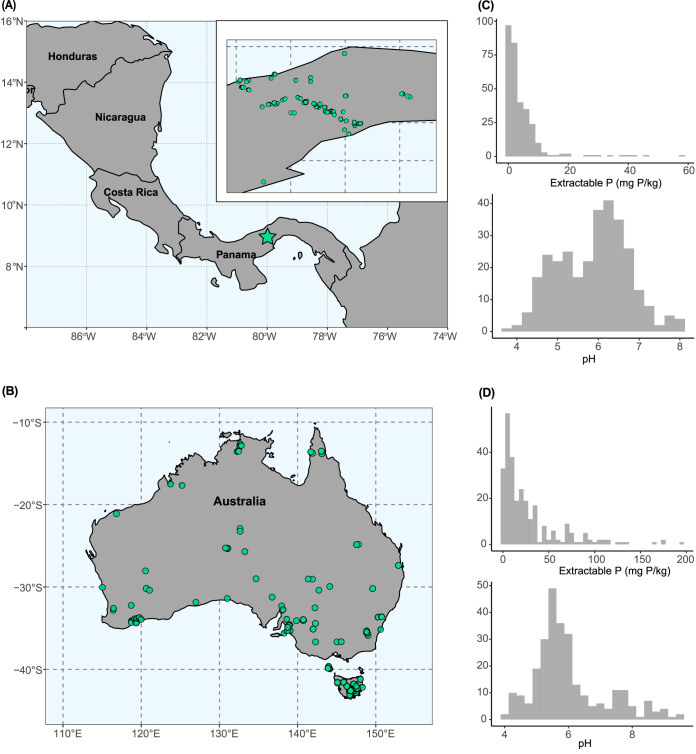
Description of the soil collection effort and the distribution of available phosphorus concentrations across all samples. (A) Panama (*n* = 308 soils for 16S rRNA gene amplicon sequencing and 92 soils for shotgun metagenomics) and (B) Australia (*n* = 275 soils for both 16S rRNA gene amplicon sequencing and shotgun metagenomics). (C) A density plot displaying the distribution of available phosphorus (log-transformed for analyses) across all Panama soils, as estimated by resin phosphorus availability. (D) The same density plot for Australia soils, as estimated by Colwell phosphorus availability (log-transformed for analyses).

Here, we sought to identify the soil microbial taxa (*Bacteria* and *Archaea*) and their metabolic traits that shift with soil P availability, given the importance of P limitation in structuring terrestrial systems. We analyzed soils from two distinct sample sets, namely, 320 soils from a series of tropical forest plots located across the isthmus of Panama and 275 soils that span diverse biome types across Australia (Fig. 1). Both systems span natural gradients in soil-soluble P concentrations but exhibit pronounced variation in other soil and site properties. Our goal was to identify how soil microbial communities respond to and cope with P limitation and whether such P responses are broadly conserved across belowground systems, information that is directly relevant for understanding microbial adaptations to P limitation and the microbial controls on soil P dynamics.

## RESULTS

### Effect of extractable P and other soil and climatic parameters on bacterial and archaeal communities.

We leveraged two independent sample sets to investigate how soil microbial communities shift across gradients in P availability (Fig. 1). Our sample sets comprised 320 soils collected in Panama (308 were retained for analyses; see Materials and Methods for field sampling details) and 275 soils from Australia collected and sequenced previously as a part of the Biomes of Australian Soil Environments project (20). Specifically, we asked which taxa and metabolic pathways were enhanced in soils that have relatively less extractable P or relatively more extractable P, which we term “low P” versus “high P” soils. Notably, different regions of the 16S rRNA gene were sequenced for the Panama and Australia soil sets. We used two separate data sets that were collected and processed independently that were processes critical to our study design, enabling us to determine whether there were consistent and generalizable patterns in microbial responses to P availability across two very different environmental gradients. The Panamanian soils are primarily from forested sites that receive 1,488 to 3,538 mm of rainfall per year, with soil pH values across this sample set ranging from 3.85 to 8.09. In contrast, the Australian soils spanned a broad range of different biome types (including grasslands, forests, and shrublands) with mean annual precipitation levels from 161 to 2,147 mm and soil pH values ranging from 4.0 to 9.6. See Tables S1 and S2 and Fig. S1 and S2 in the supplemental material for information on soil and site characteristics for all Australian and Panamanian soils, respectively. Despite the distinct nature of the two sample sets, both represented pronounced gradients in extractable soil P concentrations. Two different indices were used to estimate the amount of extractable P in soils (extractable resin P versus Colwell P [21, 22]), but both sample sets represent broad gradients in extractable P. Indices of extractable P ranged from 0.02 to 57.19 mg P kg^−1^ (resin P) across the Panamanian soil samples and from undetectable to 193 mg P kg^−1^ (Colwell P) across the Australian soils (see Materials and Methods for a description of how we estimated the availability of soil P for each sample set).

10.1128/mBio.01718-20.1 FIG S1 The pairwise Pearson’s correlations across 68 environmental predictors for the Panama sample set (*n* = 308 soils). Download FIG S1, EPS file, 1.2 MB. Copyright © 2020 Oliverio et al. 2020 Oliverio et al. This content is distributed under the terms of the Creative Commons Attribution 4.0 International license.

10.1128/mBio.01718-20.2 FIG S2 The pairwise Pearson’s correlations across 68 environmental predictors for the Australia sample set (*n* = 275 soils). Download FIG S2, EPS file, 1.8 MB. Copyright © 2020 Oliverio et al. 2020 Oliverio et al. This content is distributed under the terms of the Creative Commons Attribution 4.0 International license.

10.1128/mBio.01718-20.6 TABLE S1 Location of Australia study sites and selected environmental data. Download Table S1, XLSX file, 0.03 MB. Copyright © 2020 Oliverio et al. 2020 Oliverio et al. This content is distributed under the terms of the Creative Commons Attribution 4.0 International license.

10.1128/mBio.01718-20.7 TABLE S2 Location of Panama study sites and selected environmental data. Download Table S2, XLSX file, 0.04 MB. Copyright © 2020 Oliverio et al. 2020 Oliverio et al. This content is distributed under the terms of the Creative Commons Attribution 4.0 International license.

As expected, given the large differences in soil and site characteristics across the two sample sets, there was pronounced variation in the composition of the soil bacterial communities both within and across the Australian and Panamanian sample sets. Although the dominant classes, namely, *Alphaproteobacteria*, *Gammaproteobacteria*, *Acidobacteriia*, and *Verrucomicrobia*, are typical of most soils (23), the relative abundances of these taxa varied dramatically depending on the soil and sample set in question. For example, the mean abundance of *Acidobacteriia* was highly variable within each sample set (0% to 29% of all 16S rRNA gene reads in Panama and 1% to 44% in Australia) (see Fig. S3 in the supplemental material). Thus, we had two independent and distinct sample sets that both represent gradients in P availability but include soils with a broad range of edaphic properties, site characteristics, and prokaryotic community compositions.

10.1128/mBio.01718-20.3 FIG S3 The dominant soil bacterial taxa vary in relative abundances across the Panama and Australia sample sets. Boxplots of the relative abundances for the top 10 bacterial classes for the Panama sample set (308 soils) and the Australia sample set (275 soils). Note that no archaeal taxa were dominant enough to be included in this list. For both sample sets, classes are ranked by their mean relative abundance. Download FIG S3, EPS file, 0.7 MB. Copyright © 2020 Oliverio et al. 2020 Oliverio et al. This content is distributed under the terms of the Creative Commons Attribution 4.0 International license.

To evaluate the relative importance of P in structuring communities, we first assessed the correlation between differences in community composition and extractable P concentrations with Mantel tests. For both sample sets, soils with more similar levels of extractable P tended to share more similar bacterial and archaeal communities (the rho value of Mantel tests, e.g., r_M_ = 0.24, *P < *0.0001; and r_M_ = 0.21, *P < *0.0001; for Panama and Australia, respectively). We next asked whether extractable P levels explained a unique portion of the variation in community composition with the inclusion of other soil and climatic predictors. We assessed this question using multiple regressions on dissimilarity matrices, determining the subset of environmental predictors that best explained community dissimilarities. For both sample sets, differences in community composition were predicted by a combination of soil and climatic variables (*R^2^* = 77% and 60% for Panama and Australia, respectively; *P* < 0.0001 for all predictors) (see Fig. S4 in the supplemental material). For both models, pH was the most important predictor, although extractable P, along with other soil and climatic predictors, also explained a unique portion of the variation in prokaryotic community composition.

10.1128/mBio.01718-20.4 FIG S4 Soil microbial communities are strongly shaped by pH and to a lesser extent by other soil and climatic properties, including available phosphorus. (A, B) Nonmetric multidimensional scaling (NMDS) ordinations of Panama soils, highlighting differences in microbial community composition by predictors of community composition, including pH and available phosphorus, respectively. (C) The environmental predictors included in the best model for explaining overall variation (overall R^2^ = 77%) across Panama soil communities, ordered by importance (regression coefficients of predictors from multiple regression on distance matrices), including soil pH, manganese (Mn), available phosphorus (resin P), and dissolved organic carbon (DOC), and also by climatic properties, including mean annual temperature (MAT) and mean annual precipitation (MAP). (D, E) NMDS ordinations of Australia soils by pH and available P, respectively. (F) The environmental predictors for Australia soil communities (overall R^2^ = 60%) in order of importance, including soil pH (pH), MAT, MAP, sulfur (Sulf), net primary productivity (NPP), and available phosphorus (P). Download FIG S4, TIF file, 0.5 MB. Copyright © 2020 Oliverio et al. 2020 Oliverio et al. This content is distributed under the terms of the Creative Commons Attribution 4.0 International license.

### Identifying the microbial taxa associated with P availability.

**FIG 2:**
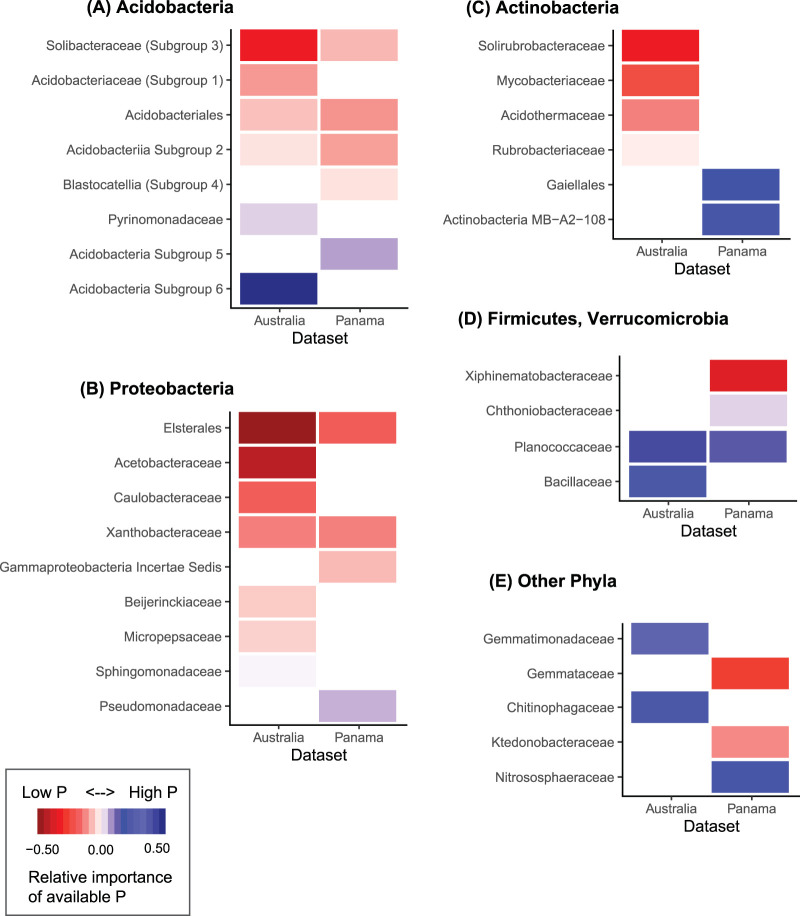
The bacterial and archaeal taxa that were significantly enriched in soils with low (red) or high (blue) P availability (A–E). Colors are scaled by relative importance of P availability to each overall environmental distribution model. For all bacterial or archaeal taxa, we assessed the importance of P availability in explaining distributions using generalized linear models (see Materials and Methods), and only those taxa that were sufficiently abundant and for which available P was retained in the final model are shown here (see Materials and Methods for details).

We next investigated which particular prokaryotic taxa were associated with P availability and to what extent we could identify shared taxa that were associated with either low P or high P soils across both sample sets, despite their unique soil and site characteristics. Of the more abundant taxa (those 27 families with mean relative abundances of ≥1% in each sample set), we identified 17 taxa in Panama and 21 taxa in Australia that were more likely to be abundant in either high P or low P soils (Fig. 2). In other words, there were a subset of bacterial and archaeal taxa that our models indicate were sensitive to P availability (Fig. 2; see Table S3 in the supplemental material). These taxa were mostly bacteria—only one group of archaea (*Nitrosphaeraceae*) were determined to be responsive to high P (only in the Panama sample set). We thus focus on bacterial responses throughout our results and discussion of microbial taxa. Of those families associated with extractable P concentrations, 14 in Australia and 10 in Panama were enriched (higher relative abundances) in soils with lower extractable P (i.e., low P taxa) while the other 7 taxa in both data sets were enriched in soils with greater extractable P (i.e., high P taxa). Six taxa overlapped (i.e., were found to be P responsive in both sample sets), with these 6 families being identified as low P (5 of 6) or high P (1 of 6). Three of those low P taxa found in both the Panamanian and Australian sample sets were within the *Acidobacteria* phylum (including *Acidobacteriia* subgroup 2, *Solibacteraceae* subgroup 3, and *Acidobacteriales*) and two within the *Alphaproteobacteria* phylum (*Xanthobacteraceae* and *Elsterales*). Generally, both the *Acidobacteria* and *Alphaproteobacteria* phyla contained many low P taxa. In both sample sets, the *Firmicutes*, including *Planococcaceae* and *Bacillaceae*, were enriched in high P soils.

10.1128/mBio.01718-20.8 TABLE S3 Relationships between abundant soil microbial taxa and available P, for the subset of taxa in which available P was included as a predictor in the best generalized linear model to explain variation in the distribution of relative abundance. The R^2^ values reported refer to overall model fit, including available P and other soil and climatic variables retained for the best models. Taxa distribution models were built at the family level in order to facilitate comparisons across datasets (see Materials and Methods for details). Download Table S3, XLSX file, 0.02 MB. Copyright © 2020 Oliverio et al. 2020 Oliverio et al. This content is distributed under the terms of the Creative Commons Attribution 4.0 International license.

We then determined how the taxa that were identified as being responsive to P availability might differ with respect to their maximum potential growth rates, a key life history trait. To do so, we obtained estimates of rRNA gene copy numbers for those taxa shown in Fig. 2 identified as being associated with soil P availability, using rRNA gene copy number as a proxy for maximum potential growth rate (24). We found that those taxa which were relatively more abundant in low P soils had a significantly lower mean copy number (and presumably lower maximum potential growth rates) than that of the high P taxa (see Fig. S5 in the supplemental material) (mean copy number of high P taxa = 3.71, low *P* = 1.99 and *P* < 0.05).

10.1128/mBio.01718-20.5 FIG S5 The estimated average rRNA gene copy number in the P-limited versus P-rich bacterial families (copy number estimated from rrnDB v5.5). Low P soils had a significantly lower mean copy number than the high P taxa (mean copy number of high P taxa, 3.71; low P taxa, 1.99; Kruskal-Wallis test, *P* < 0.05). Download FIG S5, EPS file, 0.5 MB. Copyright © 2020 Oliverio et al. 2020 Oliverio et al. This content is distributed under the terms of the Creative Commons Attribution 4.0 International license.

### Identifying gene functions and pathways associated with P availability.

We next sought to identify genomic features associated with soil P availability, focusing on those SEED functional categories putatively linked to P metabolism across prokaryotes. We opted to exclusively focus analyses on gene functions and pathways known to be involved in P metabolism to focus our hypotheses and reduce the chance of detecting spurious correlations. These P-related genes accounted for ∼1.5% of all annotated reads in each of the sample sets (analyses based on 275 Australian soil metagenomes and 92 Panamanian soil metagenomes; see Materials and Methods). The most abundant P metabolism-related genes in Panamanian and Australian soil systems were assigned to the following categories: phosphate metabolism (72.5% and 74% of all P metabolism reads, respectively), high-affinity phosphate transporter and control of PHO regulon (17% in both), and alkylphosphonate utilization (8% and 7%) (see Table S4 in the supplemental material). The most abundant specific SEED functions across both soil systems included the phosphate regulon sensor/response genes *phoR* and *phoB*, which together code for gene products involved in sensing and regulating phosphate concentrations and activate the high-affinity phosphate specific transport system *pstSCAB* when phosphate starvation is detected (25). The *phoB*, *phoR*, and *pstSCAB* components were detected in the top 10% of all SEED functions in both systems. Other relatively abundant P metabolism genes included alkaline phosphatases, low-affinity inorganic phosphate transporters, other components of the *pho* regulatory network, and components of the C-P lyase core complex, a metabolic pathway that enables bacteria to utilize organophosphonate compounds.

10.1128/mBio.01718-20.9 TABLE S4 The mean relative abundance of major SEED subsystems (level 3) within P metabolism for both Australia and Panama sample sets. Download Table S4, XLSX file, 0.01 MB. Copyright © 2020 Oliverio et al. 2020 Oliverio et al. This content is distributed under the terms of the Creative Commons Attribution 4.0 International license.

**FIG 3:**
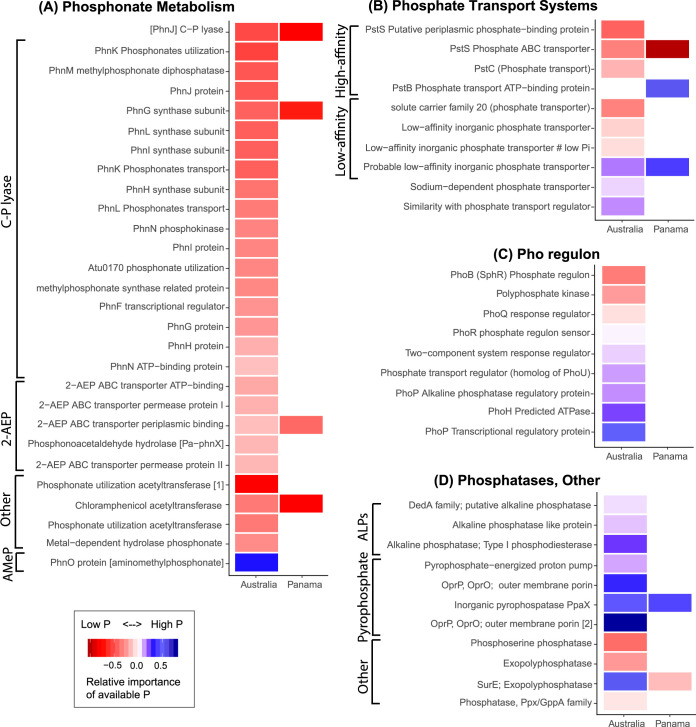
The bacterial and archaeal genes (as annotated by SEED subsystems) that were significantly enriched in soils with low (red) or high (blue) P availability. Colors are scaled by relative importance of P availability to each overall environmental distribution model. The SEED functions associated with phosphonate metabolisms, including C-P lyase (methylphosphonate degradation I), 2-AEP (e.g., 2-aminoethylphosphonate degradation I or the phosphonotase pathway), AMeP (e.g., aminomethylphosphonate degradation), and other phosphonate-associated genes (A); phosphate transport systems, including the high-affinity *pstSCAB* transport system and low-affinity inorganic phosphate transporters (B); components of the Pho regulatory network (C); and various phosphatases, including pyrophosphatases and alkaline phosphatases (ALPs) (D). We note that a small subset of genes (*n*, <15) are not visualized here due to either an unclear functional categorization or a function that does not fit in our highlighted categories. Also, some names are abbreviated—for a complete list and full names see Table S5.

As with the taxonomic analyses, we built distribution models identifying genes or gene categories that vary in relative abundance across P gradients to infer the microbial strategies or traits linked to P acquisition, running these models independently for the two data sets (see Table S5 in the supplemental material). Of the 145 SEED functions associated with P metabolism across the Panamanian sample set, our models show that the relative abundances of 11 of these SEED functions were significantly correlated with P availability (6 low P and 5 high P) (Fig. 3). Of the 195 P metabolism functions in the Australian sample set, 68 were correlated with P availability (44 low P and 25 high P) (Fig. 3). The higher rates of the detection of genes associated with P availability from the Australian soils data set are likely related to a much greater sequencing depth per sample (approximately 4.2 million versus 0.45 million annotated reads per sample, on average, for the Australian and Panamanian soils, respectively). Likewise, we obtained shotgun metagenomic data from more Australian than Panamanian soils (275 versus 92), and there was a weaker correlation between soil pH and soil extractable P across the Australian sample set (Pearson’s product moment correlation = 0.43 with a *P* value of <0.0001 for Panama versus no significant correlation across the Australian data set), making it more difficult to disentangle responses to P availability versus soil pH across the shotgun metagenomes obtained from the Panamanian samples.

10.1128/mBio.01718-20.10 TABLE S5 Relationships between abundant genes/gene functional groups related to P metabolism (annotated by SEED subsystems) and available P, for the subset of SEED functions in which available P was included as a predictor in the best generalized linear model to explain variation in the distribution of relative abundance. The R^2^ values reported refer to the overall model fit of the functional distribution model, including available P and other soil and climatic variables retained for the best models. Download Table S5, XLSX file, 0.02 MB. Copyright © 2020 Oliverio et al. 2020 Oliverio et al. This content is distributed under the terms of the Creative Commons Attribution 4.0 International license.

**FIG 4:**
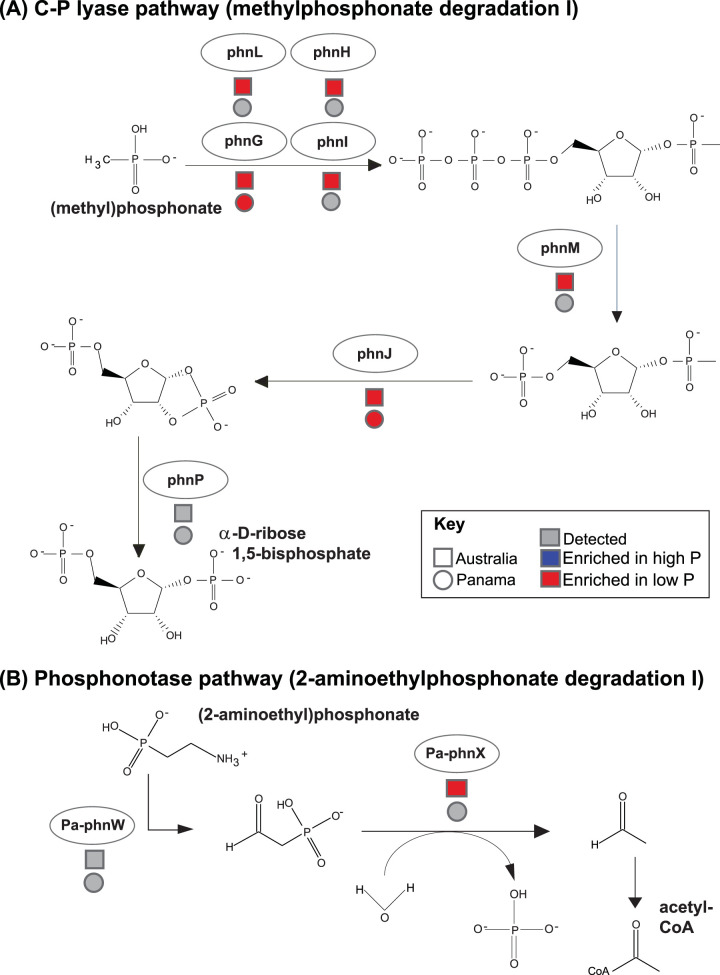
Organophosphonate utilization pathways are enriched in low P soils. (A) The core C-P lyase pathway and detection of particular components across both data sets. We note that *phnP* was not classified under phosphorus metabolism in the SEED subsystems, so we did not test for responsiveness to P availability; however, we did detect *phnP* in both data sets. (B) The phosphonotase pathway and detection of components across both data sets. For all gene categories, red indicates that a category is significantly enriched in low P soils. Squares and circles refer to detection in the Australian and Panamanian soils, respectively.

Numerous genes associated with components of the C-P lyase core reaction pathway (methylphosphonate degradation I) were linked to P availability in both data sets (Fig. 4). They included some of the core complex genes along with other components associated with the C-P lyase pathway, e.g., organophosphonate acetyltransferases and organophosphonate synthase proteins (Fig. 3 and 4). Most of these components were found to be significantly enriched in low P soils in one or both of the study systems, and the relative importance of extractable P to the overall explanatory power of the distribution model was high for many components. For example, the relative importance of extractable P for the C-P lyase (*phnJ*) distribution models was 77% and 54% for Australia and Panama, respectively (Table S5). We also detected *Pa*-*phnWX* and other functions associated with the phosphonotase pathway (2-aminoethylphosphonate degradation I), of which many were again enriched in low P soils (Fig. 3 and 4). Components of *pstSCAB*, a high-affinity, high-velocity phosphate transport system, were predominately enriched in low P soils in both the Panamanian and Australian data sets. The particular functions enriched in high P soils in both systems included inorganic pyrophosphatase (*ppaX*) and a probable low-affinity inorganic phosphate transporter (Table S5; Fig. 3). Other phosphatases were also more abundant in high P Australian soils, including alkaline phosphatase (ALP). Together, these results highlight that not only are there specific microbial taxa consistently associated with high P or low P soils (Fig. 2) but also specific genes associated with P metabolism (namely, *phn*, *pst*, and *pho* genes, Fig. 3) exhibit consistent shifts in relative abundances with soil P availability across the two independent data sets.

## DISCUSSION

We found that extractable P concentrations explain a significant portion of the variation in overall soil prokaryotic community composition in both the Panamanian and Australian soil data sets. While other climatic and edaphic characteristics were often better predictors of community composition (most notably soil pH, consistent with previous work; e.g., reference 26), P availability explained a significant portion of the variation in soil microbial community composition. These findings support other studies that have also identified P availability as an important factor shaping microbial distributions in soil (27, 28), highlighting the broad importance of P availability in shaping belowground communities across diverse ecosystems and soil types.

Beyond identifying associations between overall prokaryotic community composition and soil P availability, we were also able to identify particular bacterial and archaeal taxa which consistently exhibited shifts in relative abundances across gradients in soil P availability (Fig. 2). For example, the *Acidobacteria*, *Solibacteraceae* subgroup 3, and *Acidobacteriales* were consistently enriched in low P soils, and *Planococcaceae* were relatively more abundant in high P soils across the two study systems. Notably, *Solibacteraceae* are hypothesized to acquire phosphorus from infertile soils (29). While the specific traits and life history strategies of these P-associated taxa remain largely undetermined (primarily because so few have been well characterized), there appear to be notable differences in the presumed traits of specific bacterial taxa associated with low P versus high P soils. Our data suggest that the limited availability of soil P likely constrains microbial growth, favoring slower growing, oligotrophic microbes that can persist with lower levels of nutrient availability. *Acidobacteria* are typically classified as more oligotrophic bacteria (30, 31), whereas most *Firmicutes* and *Chitinophagaceae* (within the *Bacteroidetes* phylum) are generally considered copiotrophic (32). Our estimates of maximum potential growth rates (inferred from average rRNA gene copy number) yielded additional evidence that increasing P availability tends to select for more copiotrophic bacterial taxa. We found a higher average rRNA gene copy number in high P soils (e.g., fast growers), which is likely an advantageous trait in nutrient-rich environments that can support rapid growth (Fig. S5). We also found that *Alphaproteobacteria* were consistently enriched in low P soils, consistent with results of reference 33, but members of this phylum are not readily categorized as either copiotrophic or oligotrophic, suggesting that this lineage may have a unique competitive advantage that enables persistence in P-limited soil environments. Indeed, many *Alphaproteobacteria* have been described as plant growth-promoting microbes (PGPMs) and some taxa are thought to solubilize mineral soil P pools (34), but the specific contributions of these taxa to P solubilization across the soils studied here remains undetermined.

The P-responsive taxa we identified may serve as useful bioindicators of P availability—either to estimate differences in soil P availability across sites if those data are unavailable or to complement more traditional chemical analyses. The subset of taxa we identified as having significant, consistent responses to P availability across both data sets suggest they are sufficiently widespread and responsive to serve as bioindicators of P availability, even across a broad range of distinct soil types. For example, the *Xanthobacteraceae* were enriched in low P soils in both the Australian and Panamanian sample sets, a finding that is consistent with other recent work that identified bacterial indicators of soil conditions and found *Bradyrhizobium* (a lineage within the *Xanthobacteraceae*) to be a strong indicator of low P soils across a range of soil types (33). There is clear utility to using the relative abundances of these P-associated taxa as bioindicators of soil P availability, as they could serve as metrics of P status in natural ecosystems where conventional measures of agronomic P availability might not account for the diversity of P-cycling processes and P acquisition strategies used by plants and microbes at a given site.

Our metagenome-based characterization of P metabolism genes across the two independent sample sets suggests that there are some underrecognized metabolic strategies employed by soil bacteria to cope with P-limiting conditions. Specifically, our results suggest that, when phosphate availability is limited in soil, a major adaptive strategy employed by bacteria is to catabolize organophosphonates to acquire P by two main metabolic pathways. One is the C-P lyase pathway (e.g., methylphosphonate degradation), which has evolved to cleave phosphate from methylphosphonates (Fig. 4). The second is the phosphonotase pathway, whereby phosphate is obtained from 2-aminoethylphosphonate (2-AEP). Although organophosphonates are thought to comprise a relatively small portion of the total P pool in soils (4, 35), they are nevertheless ubiquitous in nature as constituents of lipids, polysaccharides, polypeptides, and microbial necromass (36). Indeed, the presence of 2-AEP, a key phosphonotase substrate (Fig. 4), has been isolated from the lipids of ciliates (37), one of the most abundant group of soil protists globally (38).

Our identification of P-responsive taxa also supports our conclusion that organophosphonate recycling systems are likely important strategies by which soil bacteria obtain P from infertile soils. First, components of the C-P lyase have been found in multiple *Alphaproteobacteria* (9), including *Rhizobiales* (the order which contains *Beijerinckiaceae* and *Xanthobacteraceae*, taxa we found to be enriched in more low P soils) (Fig. 2). Second, these *Rhizobiales* associated with C-P lyase pathways were also found to be prevalent in phosphate-limited marine habitats (9). Third, some of the bacterial families we identified as associated with P deficiency are comprised of taxa with known methanotrophs (including *Beijerinckiaceae*). These bacteria could be sustained by the methane released from the methylphosphonate pathway (39), potentially explaining their success in P-limited soils. While we do not know which taxa are directly responsible for organophosphonate utilization in low P soils, the taxa we found to be enriched in low P soils are worthy of more targeted investigations.

Our results are supported by previous work highlighting the importance of organophosphonate recycling systems in low P soils. First, bacteria isolated from soil can utilize both methylphosphonate (MPn) and 2-AEP as sole sources of P *in vitro* (40, 41). Thus, in addition to having the genes and metabolic pathways necessary to obtain P from organophosphonate compounds, some soil bacteria can use these compounds as their sole P source. Second, bacterial utilization of MPn was shown to be suppressed in the presence of orthophosphate (39, 42), suggesting that organophosphonate use occurs when more readily available forms of P are limited. While bacterial isolates from soil have been shown to have the capacity to use a variety of organophosphonates (11, 40, 43), the importance of this process in soil remains undetermined. However, a few studies have detected phosphonotase activity in soil and suggest that microbial degradation of phosphonates is favored in P-limited soils (11, 18). Finally, work conducted in P-limited marine environments has also shown that C-P lyase genes are abundant in a range of bacterial taxa (9, 44). Thus, in both marine and soil environments, methylphosphonate degradation may represent an important strategy for microbial survival in P-limited conditions.

In addition to organophosphonate utilization, our data suggest that bacteria in low P soils may have particular strategies to permit efficient phosphate uptake. The phosphate regulon (Pho) is a regulatory mechanism involved in the sensing and regulation of available phosphate, controlled by a two-component regulatory system known as *phoR*-*phoB* in Escherichia coli (with a variety of other names and associated response regulatory components in other bacteria) (45). Under P-limited conditions, *phoB* is activated by *phoR*, while under high P conditions, *phoU* is required for *phoB*. This observed response appears to be consistent with our findings; *phoB* was enriched in low P soils, while *phoU* and *phoR* were more abundant in high P soils (Fig. 3). In a comparison of metagenomes from two temperate forest soils (18), a significantly greater abundance of *phoR* genes was also detected in the high P soil. However, the complete signal transduction system that senses phosphate scarcity has yet to be fully described (45). The high-affinity phosphate-specific transporter Pst system is a highly conserved component of the Pho regulon in bacteria (e.g., the *pstSCAB* systems). We found that *pstS* was abundant in low P soils (Fig. 3). In E. coli, this system is induced in response to P starvation (46). Furthermore, *pstS* has been observed to track P availability via differential expression compared to *pstCAB* (47, 48). These P-specific regulatory and transport systems likely represent adaptive strategies used by soil bacteria to overcome P limitation in soils.

In contrast to P-limited soils, a significantly higher microbial potential for utilization of inorganic P was found in high P soils, including *ppaX* (inorganic pyrophosphatase), which was enriched in high P soils across both data sets. Our observation of a greater potential for inorganic P solubilization in high P soils is consistent with a previous metagenomic-based study of soils from two forested sites (18) and the abundance of dissolved inorganic pyrophosphate in soil solution across the P gradient in Panama (49). We also detected more SEED functions corresponding to alkaline phosphatases (ALPs) in high P soils. Alkaline phosphatase activity is often detected in P enriched environments (50, 51) but is typically detectable only at a relatively high pH—which might therefore be linked to the weak P-pH relationship (52). It is also important to note that there are likely other genes and pathways not directly classified under P metabolism that may contribute to various aspects of P cycling. For example, the abundance of phytase genes at low P suggests that the degradation of phytate provides an important source of P at low fertility (15). In addition, there are likely many genes and pathways related to phosphorus metabolism that have not yet been characterized and were not captured with our analytical approach. As functional gene annotations continue to improve, we will likely identify additional strategies used by microbes to cope with P scarcity in soil systems.

By comparing two independent sample sets (soils from Australia and Panama) that each capture gradients in P availability but differ in many other important ways, this work establishes a broader understanding of the strategies that soil microbes employ across gradients in P availability. Our lineage-based analyses suggest that low P soils generally harbored more oligotrophic taxa, while high P soils were comprised of more copiotrophic taxa. However, these proposed shifts in life history strategies were inferred from taxonomic information and from differences in average rRNA gene copy number (a proxy for maximum potential growth rates) (53). Thus, these findings need to be confirmed with additional evidence given that the traits of most soil microbial taxa, including those identified here as being P responsive, remain largely uncharacterized. Our results also suggest that phosphonate utilization by bacteria is a key adaptive strategy they use to cope with highly P-limited soil conditions. These findings build on studies conducted in marine systems by highlighting that phosphonate utilization is likely a key strategy used by bacteria to cope with P scarcity in both terrestrial and marine systems. Although soil phosphonate pools are difficult to quantify (54), our results suggest that measuring *in situ* rates of soil phosphonate turnover would improve our understanding of soil P dynamics and how microbes cope with the P-limited conditions that are so common in soils across the globe.

## MATERIALS AND METHODS

### Panama field sampling.

We collected 320 mineral soil samples (0 to 10 cm) after removing litter from 64 forest census plots in Panama. Samples were collected during the wet season between July and December 2017. At each site, 5 soil cores (0 to 10 cm) were collected (64 sites by 5 soils per site = 320 unique soils). Each soil sample was a composite of multiple 1-inch cores from an area of approximately 30 by 30 cm. The 64 sites span a marked gradient of phosphorus availability across a gradient in mean annual precipitation (from 1,488 to 3,538 mm) and represent a broad range in soil properties (Table S2). After field collection, all soils were separated into two subsamples—the first subsamples were immediately stored at −20°C until DNA extraction, and the second subsamples were air dried for soil chemical analyses. Soil extractable Al, Ca, Fe, K, Mg, Mn, and Zn were determined by Mehlich-III extraction (55). Total C and N were determined by dry combustion. Resin P was determined by extraction with anion-exchange membranes (56). We consider the resin P procedure to provide a sensitive measure of soil P availability that has ecological significance, as it is associated strongly with tree species distributions and growth in lowland and montane forests (6, 57–59).

Latitude and longitude were recorded at each sample location and used to obtain information on a range of climatic and surface variables (23). We obtained information on mean annual temperature, mean annual precipitation, precipitation seasonality, mean diurnal range, maximum and minimum temperature, and aridity index from the WorldClim database (60), with the Normalized Difference Vegetation Index (NDVI) serving as a proxy for net plant primary productivity (61).

### 16S rRNA gene and shotgun metagenomic sequencing.

To characterize the bacterial and archaeal communities from the 320 Panama soils, we extracted DNA and then amplified a 250-bp fragment of the V4-V5 region of the 16S rRNA gene as described in reference 62. In brief, a sterile swab was inserted into each sample, and we extracted DNA from the swabs using the PowerSoil DNA extraction kit (Qiagen). After extraction, we PCR amplified each DNA sample in duplicate reactions using the 515f/806r primers that included the appropriate Illumina adapters and 12-bp barcodes to enable multiplexed sequencing (63). Amplicon concentrations were normalized and sequenced on the Illumina MiSeq platform with 2 × 150-bp paired-end chemistry at the University of Colorado Next Generation Sequencing Facility. We sequenced multiple negative controls per plate to check for possible contamination.

Raw sequence data were processed as amplicon sequence variants (ASVs) with the DADA2 pipeline (64), as implemented in https://github.com/amoliverio/dada2_fiererlab. The DADA2 pipeline resolves ASVs rather than clustering sequences by percent identity. Briefly, sequences with unassigned bases (e.g., Ns) were removed prior to primer removal, and then sequences were quality filtered with the following parameters: truncLen = 150 for forward reads and 140 for reverse reads, maxEE = 1, and truncQ = 11. After quality filtering, sequence variants were inferred with the DADA2 algorithm, and then we merged paired-end reads. Next, we removed chimeras and assigned taxonomy as per the DADA2 pipeline, using the SILVA database v132 (65) with the RDP naive Bayesian classifier (66). We filtered the table to remove any reads that were assigned as chloroplast, mitochondria, or eukaryotes and likewise removed reads that were unassigned at the phylum level. ASVs were retained if they were comprised of at least 10 reads across the data set. We set a minimum coverage threshold of ≥8,000 reads per sample, and 12 samples were discarded due to insufficient sequence coverage, leaving 308 samples for downstream analyses. We normalized the sample sequence counts by cumulative-sum scaling (67).

We also characterized a subset of 92 Panama soils via shotgun metagenomic sequencing. We selected 2 to 3 soils from each of 45 sites. We prepared the samples for shotgun metagenomic sequencing as per reference 38, and samples were sequenced on the Illumina NextSeq platform with 2 × 150-bp chemistry at the University of Colorado Next Generation Sequencing Facility. We filtered raw reads with Sickle (68) with the specified parameters -q 50 and -I 20. To obtain functional annotations of reads into hierarchies of SEED functions within pathways, we used the Superfocus pipeline (69) with SEED subsystems (70), which annotates reads of prokaryotic origin (note that we cannot determine the taxonomic affiliations of individual reads). We normalized the annotated table (all 92 samples had >194,590 annotated reads per sample) via cumulative-sum scaling (67).

### Australia soil microbial community data sets.

We used a subset of soils from the Biomes of Australian Soil Environments (BASE) project (20). The 275 soil samples we selected spanned a wide range in site and soil properties (Table S1). Soils were all collected and sequenced according to the methods described in Bissett et al. (20). In brief, at each sampling location, a composite soil sample was collected from nine soil samples from the top 0 to 10 cm. One subsample was immediately frozen for DNA extraction (−20°C) and the other was air dried for chemical analyses. Soil properties included texture, pH, conductivity, total organic C, available N, available P, available K, and total Al, B, Ca, Cu, Fe, K, Na, Mg, Mn, S, and Zn (20). Climatic variables, including mean annual precipitation and temperature, were obtained as described above for the Panama data set.

Soils were extracted in triplicate as per methods employed by the Earth Microbiome Project (71). For 16S rRNA gene amplicon sequencing, the extracted DNA was amplified with the 27F-519R region, and raw sequence data were processed into amplicon sequence variants (ASVs) with the BASE pipeline (20). We used the DADA2 pipeline, as described above, to assign taxonomy with the SILVA database so that taxonomic assignments would be comparable with those used for the Panama data set. We then filtered the ASV table, as per the Panama data set, to include only ASVs that were represented by at least 10 reads across the data set. We set a minimum coverage threshold of ≥4,000 reads per sample and normalized the ASV table via cumulative-sum scaling, as described above.

We also obtained shotgun metagenomic data for the same set of 275 Australian soils. Methods for shotgun metagenomic library preparation and sequencing are described on the BASE methods website (https://data.bioplatforms.com/organization/pages/australian-microbiome/methods). In brief, 200 ng of genomic DNA was sheared to 550 bp using the Covaris system. Fragments were then purified using the AMPure system, and libraries were prepared according to the TruSeq Nano DNA sample preparation guide (part no. 15041110 Rev. B, November 2013). Libraries were quantified using the Qubit high-sensitivity (HS) assay, pooled to equimolar amounts, and sequenced using HiSeq 2500 Rapid v2 kits according to the Illumina HiSeq rapid sequencing by synthesis (SBS) kit v2 reference guide (part no. 15058772, Rev. B, May 2015). The resulting sequence data were demultiplexed using Casava 1.8.2 and processed with the same annotation pipeline as described above for the Panama metagenomic samples. The gene annotation table was normalized as described above (we obtained ≥1,747,145 annotated reads from each of the 275 samples).

### Statistical analyses.

All statistical analyses were performed in R. As many of the soil and climatic variables were correlated (Fig. S1 and S2), we reduced collinearity prior to our analyses by identifying highly correlated variables and excluding those with Pearson’s *r* of <0.6. We log-transformed variables where appropriate and standardized variables prior to analyses. For the Panama data set, our final suite of 13 variables included the soil variables soil pH, available phosphorus (resin P), sulfur, total C, Al, Fe, K, Mn, dissolved organic carbon (DOC), and Ca:Mg; and the site variables mean annual precipitation, mean annual temperature, and net primary productivity. For the Australia data set, our final suite of 9 predictors included the following: soil pH, available P (Colwell), organic C, Al, Mn, sulfur, mean annual precipitation, mean annual temperature, and net primary productivity. As these soil properties were often highly variable within sites, all soils were treated independently for analyses of both the Panama and Australia data sets.

To confirm whether available P explained a unique portion of the variation in soil bacterial community composition beyond the explanatory power of other soil and climatic variables, we built models of community composition dissimilarity for both the Panama and Australia soil systems, using multiple regression on distance matrices (MRM) (72). We calculated Bray-Curtis dissimilarities using square-root-transformed relative abundances to represent differences in community composition for both data sets. In both models, MRM was implemented with Spearman correlations, the best models were derived by including all climatic and soil variables, and then we used backward elimination until all predictors explained significant variation in community composition dissimilarity. The dissimilarities in community composition were visualized with nonmetric multidimensional scaling (NMDS) ordinations.

In addition to evaluating the community-level response to P, we also assessed which, if any, particular soil bacterial and archaeal taxa were associated with P availability. To do this assessment, we built taxon distribution models with soil and climatic predictors. We chose to model the distributions of taxa at the family level because different regions of the 16S rRNA gene were sequenced for the Panama and Australia soil sample sets, which meant that comparisons at the ASV level were not possible. In addition, since many of the ASVs could not be confidently assigned to the species or genus level of taxonomic resolution, comparing data sets at finer levels of taxonomic resolution was not feasible. We only included families that were reasonably abundant (comprised a mean relative abundance of ≥1% of data set; *n* = 27 families for both data sets).

We investigated which bacterial and archaeal taxa were responsive to available P using generalized linear models (glms). In our models, we evaluated the fixed effects of the soil and climatic variables to explain the distribution of taxa across soils. Model selection was performed by first building a global model with all potential predictors and then using Akaike information criterion (AIC) values with a genetic algorithm and limiting the model search space to main effects with the glmulti package (73) to screen candidate models. For those taxa for which available P was retained in the candidate model, we built a final model. We considered a lineage to be P responsive if available P was significant as a fixed effect in the final model, which indicates an overall response. We used the model estimates for each lineage where available P was significant to specify taxa as either high P if the estimate was positive (which indicates an increase in the relative abundance with increasing levels of available phosphorus) or low P if the estimate was negative (indicating a decrease in abundance with increasing P). To assess the relative importance of predictors for models (e.g., % of model *R^2^* attributed to each predictor), we used the relaimpo package (v2.2) (74) with the “lmg model.”

To estimate the maximum potential growth rate for taxa associated with low P versus high P soils, we obtained estimates of rRNA gene copy number from the rRNA Database (*rrn*DB v5.5) (75). For each lineage, we averaged the reported copy number for all taxa classified within a family (or the lowest corresponding taxonomy for which a copy number estimate was available—importantly, for some taxa this was at the phylum level). We then determined whether the mean difference in copy numbers between families enriched in low P versus high P soils was significant at the alpha level of a *P* value of *≤*0.5. with a Mann-Whitney Wilcoxon signed-rank test.

To assess whether differences in particular gene functional abundances (as annotated by SEED functions and subsystems) were predicted by available P beyond the explanatory power of other soil and climatic characteristics, we built SEED function distribution models. Note that we did not perform any additional manual curation of SEED functions. We opted to exclusively focus analyses on gene functions and pathways known to be involved in P metabolism, as classified by the SEED annotations. They included several categories within P metabolism, as follows: high-affinity phosphate transporter and control of PHO regulon, alkylphosphonate utilization, P uptake (*Cyanobacteria*), phosphate metabolism, phosphoenolpyruvate phosphomutase, and organophosphonate metabolism. To do this analysis, we filtered our annotation tables to include only functional roles associated with P metabolism, designated at the subsystem level one, restricting downstream analyses to those genes which were detected in ≥10 soils and ≥18 soils in Panama and Australia, respectively.

To winnow down the large number of potential SEED functions, we first calculated Spearman rank partial correlation coefficients (“ppcor” package) including all variables and filtered out seed functions in which available P did not predict a unique portion of the variation in the distribution of gene relative abundances (*P* ≤ 0.05; and correlation coefficient of available P of ≥0.2). For those SEED functions that passed this conservative screening threshold, we built generalized linear distribution models as described above, first building a global model and then using the automated model selection with the glmulti package (73), which compares AIC values to come up with a best model for each SEED function. We again calculated the relative importance of predictors in all models as described above. When mapping SEED components on phosphonate pathways (Fig. 4), we considered a particular step in the pathway to be “present” if we detected either the associated subunit or protein sequence. For example, for phnG in Australia, we detected both “alpha-d-ribose 1-methylphosphonate 5-triphosphate synthase subunit PhnG” and also “PhnG protein,” and either would count as phnG detection in our pathway (e.g., filled in circle, Fig. 4). For clarity, we show all products detected that are potentially related to methylphosphonate degradation (Fig. 3).

### Data availability.

The raw sequence data for the Panama soils have been deposited in the Sequence Read Archive under BioProject accession no. PRJNA561104 and online at https://data.bioplatforms.com/organization/about/australian-microbiome. We have deposited associated data files to Figshare (https://doi.org/10.6084/m9.figshare.9977372.v1).
