# Supplementary figures and images for: The Role of Phosphorus Limitation in Shaping Soil Bacterial Communities and Their Metabolic Capabilities
Source: mBio. 2020 Oct 27;11(5):e01718-20. doi: 10.1128/mBio.01718-20 (PMC7593963; doi:10.1128/mBio.01718-20)

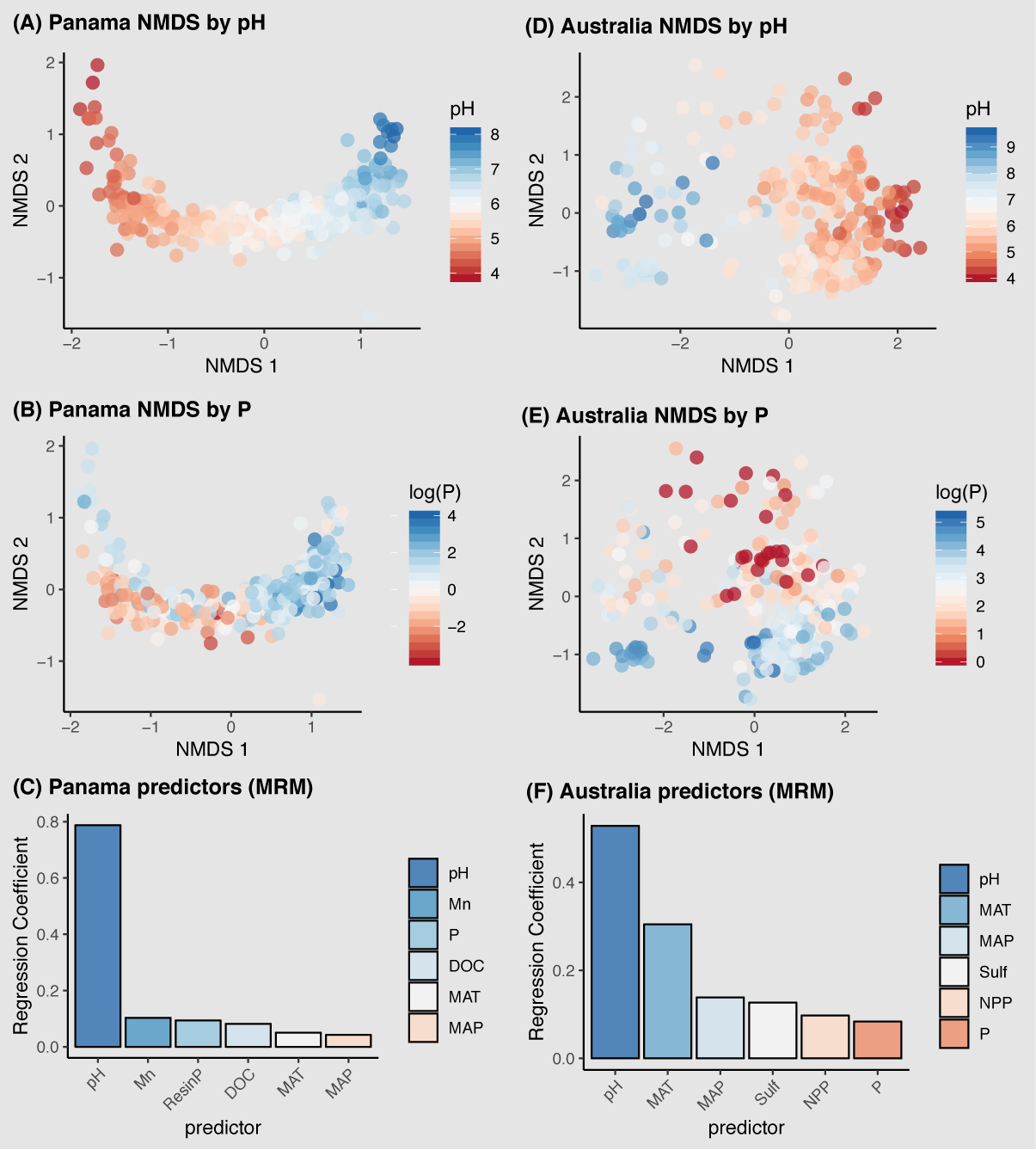

Supplement: FIG S4 [file mBio.01718-20-sf004.tif]
